# Redeployment and changes in working patterns of healthcare workers during COVID-19 in the UK: a qualitative study

**DOI:** 10.1186/s12913-025-12389-2

**Published:** 2025-02-18

**Authors:** Zainab Zuzer Lal, Mayuri Gogoi, Irtiza Qureshi, Amani Al-Oraibi, Jonathan Chaloner, Padmasayee Papineni, Susie Lagrata, Laura B. Nellums, Christopher A. Martin, Katherine Woolf, Manish Pareek, on behalf of the UK-REACH Study Collaborative Group, Katherine Woolf, Manish Pareek, Laura Gray, Laura Nellums, Anna L. Guyatt, Catherine John, I. Chris McManus, Ibrahim Abubakar, Amit Gupta, Avinash Aujayeb, Bindu Gregary, Rubina Reza, Sandra Simpson, Stephen Zingwe, Keith R. Abrams, Martin D. Tobin, Louise Wain, Sue Carr, Edward Dove, Kamlesh Khunti, David Ford, Robert Free

**Affiliations:** 1 Department of Respiratory Sciences, University of Leicester, University Road, Leicester, UK; 2 Development Centre for Population Health, University of Leicester, Leicester, UK; 3 Lifespan and Population Health, School of Medicine, University of Nottingham, Nottingham, UK; 4 The Nottingham Centre for Public Health and Epidemiology, University of Nottingham, Nottingham, UK; 5 College of Population Health, University of New Mexico, Albuquerque, NM USA; 6 University College London Medical School, London, UK; 7 Department of Infection and HIV Medicine, University Hospitals of Leicester NHS Trust, Leicester, UK; 8 National Institute of Health Research (NIHR) Leicester Biomedical Research Centre (BRC), Leicester, UK; 9 National Institute of Health Research (NIHR) Applied Health Collaboration (ARC) East Midlands, Leicester, UK; 10 Ealing Hospital, London North West University Healthcare NHS Trust, Southall, UK; 11 University College London Hospitals NHS Foundation Trust, London, UK

**Keywords:** NHS, Healthcare, Staffing, Redeployment, Ethnicity, COVID-19, Speaking up, Staff retention

## Abstract

**Background:**

Redeployment was critical in addressing the increased demands of COVID-19 on the healthcare system. Previous research indicates that ethnic minority healthcare workers (HCWs), those on visas, and in junior roles, were more likely to be redeployed to COVID-19 duties compared to White UK-born HCWs. There is limited evidence on how redeployment was practically organized, preparedness of HCWs and the NHS for rapid changes, and the decision-making processes involved. This paper discusses HCWs’ redeployment experiences, their alignment with NHS policy for deploying staff safely, and potential links to staff attrition.

**Methods:**

As part of the United Kingdom Research study into Ethnicity And COVID-19 outcomes among Healthcare workers, we conducted a qualitative sub-study, between December 2020 and July 2021, consisting of interviews and focus groups with 164 HCWs from different ethnicities, genders, job roles, migration statuses, and UK regions. Sessions were conducted online or by telephone, recorded, transcribed and analysed with participants’ consent. We utilised the breadth-and-depth approach, initially identifying transcripts mentioning redeployment or changes in work patterns, followed by an in-depth thematic analysis.

**Results:**

Of the 164 HCWs, 22 (13.4%) reported redeployment to a new role, while 42 (26.8%) reported changes in their working patterns. Redeployment experiences varied based on HCWs’ workplaces, skillsets, input into decisions, and perceived risks. Four themes were identified: 1. redeployment and the changing nature of work, 2. pandemic (un)preparedness, 3. redeployment decision-making, and 4. risk assessments in the context of redeployment. Our data revealed the practical realities of redeployment, including discrepancies between the NHS policy and actual practices, particularly early deployment without adequate training and supervision. The lack of planning and preparedness had an operational and emotional impact on HCWs, affecting their morale. Lastly, some HCWs felt disempowered and undervalued due to a lack of agency in redeployment decisions.

**Conclusion:**

This study highlights HCWs’ redeployment experiences during COVID-19, the conditions under which it occurred, and its impacts. The findings, although rooted in the pandemic, remain relevant for addressing staffing challenges in the healthcare workforce. We recommend future redeployment strategies prioritise HCWs' training and supervision, ensure strategic planning with clear communication and support for all staff, foster a sense of value among HCWs, and integrate an intersectional equity lens into workforce planning to improve staff retention and morale.

## Background

The declaration of COVID-19 as a global pandemic in March 2020 by the World Health Organisation (WHO) led to large-scale changes across several sectors – in particular the healthcare sector [1]. During the pandemic, various strategies were implemented to address the escalating demands of COVID-19-related hospitalisations. In the UK, these measures included, but were not limited to, redeployment, which involved mobilizing staff from different specialties, pay ranges or bands, and job titles or grades to support clinical areas; altering rosters and working patterns; inviting final-year medical students to apply for provisional registration from the General Medical Council (GMC); setting up temporary hospitals; inviting retired NHS staff to work on the frontline; and providing temporary Nursing and Midwifery Council (NMC) registration for internationally trained nurses [2–6]. The duration of redeployment varied and was determined locally, depending on the specific needs of the ward or the context of the situation. All these strategies, understandably, put huge demands on the healthcare workforce and raised important questions about HCWs’ well-being, exposure to risk, and fairness and parity around workforce planning and redeployment during this time [7]. Despite playing a critical role in crisis management, research suggests that redeployment significantly impacted staff well-being, with nurses being nervous about their new roles and not having a choice about the way it was implemented, and some studies claiming it was inconsistently conducted across different ethnic groups [8–10], while others found no ethnic differences [11].

HCWs from ethnic minority backgrounds experienced high mortality rates, and a disproportionately higher risk of contracting COVID-19, with poorer health outcomes when infected [10, 12]. Despite HCWs being at higher risk, empirical evidence showing how risk assessments were conducted and experienced are varied. One study found that HCWs from ethnic minority backgrounds were less likely than their White colleagues to receive risk assessments before being redeployed [13]. Conversely, our UK-REACH survey data suggest that while these HCWs were offered risk assessments, they were less likely to have changes made to their working patterns [14]. Our qualitative study revealed varied experiences of risk assessments: some participants were satisfied, while others criticised the process as merely a “tick-box” exercise. Participants also reported risks stemming from understaffing and inappropriate redeployment [15].

HCWs were more likely to be redeployed if they were nurses, particularly from ethnic minority backgrounds [10], or working on visas [11], with these characteristics potentially overlapping or occurring concurrently [8, 10]. Our analysis of redeployment-related UK-REACH survey data revealed that migrant HCWs on the Agenda for Change pay scale had 25% higher odds of being redeployed compared to those born in the UK [10]. Research suggests that these inequalities in redeployment may be due to minoritised HCWs feeling unable to challenge decisions regarding redeployment to COVID wards due to fears of repercussions or concerns about their visa status among migrant HCWs [10, 16].

Redeployment is a complex process and various factors such as training, supervision, staff competencies, and availability of personal protective equipment (PPE) have an impact on how effective it is [17]. Despite being a significant part of COVID-19 workforce restructuring, there is not enough evidence on most aspects of redeployment, except staff training needs and safety and the majority of this includes evidence from clinical HCWs only [17]. Whilst the existing literature offers valuable insights into some of the practices around HCWs’ redeployment, further information is needed to understand how other aspects of redeployment such as planning, decision-making and preparedness were experienced by HCWs on the ground and how these experiences influenced HCWs perceptions of their work and employment conditions. In-depth understanding of how HCWs experienced redeployment, the strategies redeployed staff found helpful, their perceived unmet needs during redeployment, and how these unmet needs impact staff morale are crucial for informing workforce planning. These insights are especially timely as the NHS continues to grapple with a COVID backlog exacerbated by a severe staffing crisis [18, 19]. Due to ongoing staffing shortages, HCWs are still frequently redeployed or moved to understaffed areas, leading to overwork, feelings of disempowerment, and a lack of agency [20]. These challenges are further worsened by pay erosion, ultimately resulting in staff attrition, reduced quality of care, and diminished job satisfaction [19].

In this paper, we analysed qualitative data collected in the UK-REACH project to: (a) gain a comprehensive understanding of HCWs (including non-clinical workers) redeployment experiences during the COVID-19 pandemic and; (b) to explore how these experiences reflect in practice the NHS policy for safely deploying staff during the pandemic [21] and (c) finally, how these experiences impacted staff perception about their jobs and employment. In our study, HCWs are both clinical and ancillary staff from ethnic minority and White backgrounds working in healthcare settings (e.g., front-line HCWs, ancillary staff working in hospitals, and community practitioners) [22]. The study was conducted within the UK healthcare system, which uses a structured classification system to define pay scales or bands and grades or roles. Bands refer to the NHS Agenda for Change pay structure for non-medical staff, ranging from Band 1 (entry-level) to Band 9 (senior leadership roles). Grades or job roles indicate seniority and training levels for medical and dental staff, such as junior doctors, specialty doctors, and consultants [23].

## Methods

### Study design, setting, and population

UK-REACH is a project consisting of several sub-studies with the overarching aim of evaluating the impact of COVID-19 on ethnic minority healthcare workers [24]. Data for this work come from the qualitative work stream [22]. In this study, HCWs aged 16 years and older, from both clinical and non-clinical roles, who had experience of working within healthcare settings in the UK (including all four nations – England, Scotland, Wales, and Northern Ireland) during the pandemic were eligible to take part. Due to travel and social distancing restrictions at the time, data collection, recruitment, consent, interviews, and focus groups, were conducted online remotely.

Ethical approval for the study was granted by the Brighton & Sussex Research Ethics Committee of the Health Research Authority (Ref No 20/ HRA/4718).

### Recruitment and data collection

As detailed in the study protocol [22], participants were recruited through email invitations and advertisements distributed via collaborator and partner organisations such as the Health and Care Professions Council (HCPC), General Medical Council (GMC), General Optical Council (GOC), British Association of Physicians of Indian Origin (BAPIO), Filipino Nurses Association UK (FNAUK), SERCO (sub-contracted company to carry out facilities management services*)*, and others. Recruitment and data collection took place between December 2020 and July 2021. To ensure representation across different job roles, pay ranges and UK regions, we also made targeted recruitment efforts with the help of our partner organisations. Additionally, the study was also promoted on social media to increase recruitment.

Prospective participants registered through an online interface and received a Participant Information Sheet (PIS) outlining the study. Interested participants provided online consent and completed a brief demographic questionnaire that collected information on age, gender, ethnicity, job role, job location or region of the UK, and country of birth. From amongst the pool of interested participants, purposive sampling guided the recruitment of the final sample to account for diversity in ethnicities, job roles, job location etc. Potential participants were invited to participate in either a semi-structured interview or a focus group, depending on their preference. Utilising both interviews and focus groups allowed for a comprehensive exploration of personal experiences and shared insights [25]. Semi-structured interviews facilitated detailed personal accounts, while focus groups enabled us to explore common experiences, similarities, and differences. We also considered that participants might find it easier to share sensitive information in interviews, whereas focus groups could offer broader perspectives [26].

A topic guide was developed with inputs from the Professional Expert Panel (PEP) and the Stakeholder Advisory Group (STAG), UK-REACH’s Patient and Public Involvement and Engagement (PPIE) and stakeholder groups respectively. Members of the STAG group were from the General Medical Council (GMC), Nursing and Midwifery Council (NMC), General Dental Council (GDC), General Pharmaceutical Council (GPC) and other professional bodies in the UK as detailed in our protocol [24]. The PEP group, composed of HCWs from a range of ethnic and occupational backgrounds, provided feedback on UK-REACH study materials and sampling methodology. The STAG group engaged in every stage of the UK-REACH project to provide real-world relevance to the research and its findings [24].

The topic guide explored various aspects of HCWs’ experiences during the pandemic, including fears, concerns, perceived risks, challenges faced, and supportive factors. The topic guide was piloted with the initial eight participants and while the broad topics were fixed, probe questions were loosely structured to allow researchers to explore new areas of interest as data collection progressed. Interviews lasted between 45 and 60 min, while focus groups took approximately 1.5 h, with group sizes ranging from two to seven members. Participants received a gift voucher as a token of appreciation for their contribution to the research.

Interviews and focus groups were conducted either via Microsoft Teams or by telephone (for interviews only) by trained qualitative researchers (MG, FW, AAO, LBN, OH, IQ) with experience of working with culturally and ethnically diverse communities. The interviewers introduced themselves based on their university research roles and had no prior relationship with participants prior to study commencement. With participants’ prior permission, interviews and focus groups were recorded using the Microsoft Teams recording feature or an encrypted recorder for telephone interviews, while ensuring no one else was present besides the participants and researchers. Recordings were transcribed by professional transcribers, anonymised, and checked for accuracy by the research team. Field notes were made after the interviews and focus groups.

### Data analysis

We utilised a two-step approach to analyse the transcripts, adapted from the breadth-and-depth method designed for managing and analysing large qualitative datasets. This method was particularly suitable for our research because it enabled us to systematically examine extensive data while maintaining the depth and richness of qualitative insights [27, 28]. Given that our study did not focus solely on redeployment experiences, the dataset included a broad range of participant narratives, not all of which directly addressed redeployment. However, the breadth-and-depth approach allowed us to meaningfully identify and analyse relevant data within a large sample, providing a foundation for answering our research question.

The breadth-and-depth method is a structured yet flexible qualitative methodology that balances a broad, systematic review of data with in-depth, detailed analysis of specific themes. It is particularly useful when working with large datasets, as it enables researchers to identify overarching patterns (breadth) while preserving contextual detail and complexity of social processes (depth). Further, this method allows for flexibility between theory and data-driven analysis, making it well-suited for studies like ours, where the research question was identified from a subset of the data [29].

We used Microsoft Word and Excel to conduct our analysis. 

In the initial step, we reviewed the breadth of data paying attention to keywords such as redeployment, risk assessment, change in work patterns, training, supervision (list of keywords mentioned in Appendix 1). Two researchers (MG & ZL) reviewed all the transcripts and coded sections where HCWs spoke about experiences of redeployment or changes in working patterns. This approach helped us identify broad surface-level themes that were relevant to our research question. Individual coding was discussed, and differences were resolved after thorough discussions. The coding framework and preliminary themes were then shared with the whole team. All researchers iteratively refined the framework as they reviewed transcripts, extracting rich, detailed insights.

Following the breadth analysis, the researchers conducted a more in-depth thematic analysis of the transcripts. This step focused on exploring the “how” and “why” of redeployment experiences, for example, where HCWs were either redeployed or witnessed changes in their work patterns or discussed their observations of redeployment practices in their workplaces in generic terms [30, 31]. The team met fortnightly to discuss the merging and interpretation of codes until consensus was reached on the final set of themes. Additionally, the themes were presented to the STAG members and the wider research team, with revisions made based on their feedback.

### Reflexivity

The research team was composed of a diversity of backgrounds, ethnicities including South Asian, Middle Eastern and White, migrants and UK-born, and both males and females. Their professional and academic backgrounds include dentistry, public health, social work, education, anthropology, pharmacy and health policy. Prior to data analysis and coding, team members conducted and implemented the process of reflexivity to identify and mitigate against unconscious biases [32].

## Results

The UK-REACH study recruited 164 participants and conducted 103 semi-structured interviews and 16 focus groups (see Table 1 for participant demographic data). Upon reviewing the transcripts, we found that 22 (13.4%) participants explicitly mentioned redeployment to a new role while a significant proportion (*n* = 42, 26.8%) shared experiences of changing work patterns due to the dynamic and rapidly evolving nature of the pandemic. These changes were not merely due to enhanced infection control measures mandated by all workplaces, but included significant changes to working tasks such as increased patient-facing roles, assisting in busy wards, and changes to rotas.

**Table 1:** Participant demographic data

Variable	N (%)
**Sex**	
Male	63 (38.4%)
Female	101 (61.6%)
**Age, median (IQR)**	42 (32–53)
**Ethnicity**	
Asian	65 (39.6%)
Black	29 (17.7%)
Mixed	15 (9%)
White^a^	49 (30%)
Other	6 (3.7%)
**Job Role**	
Doctors	44 (26.8%)
Nurses & Midwives	30 (18.3%)
Allied Health Professionals (AHP)^b^	62 (37.8%)
Ancillary Health Workers (AHW)^c^	28 (17.1%)
**Country of Birth**	
UK	92 (56.1%)
Outside the UK	70 (42.7%)
Missing	2 (1.2%)
**Years lived in the UK (for those born outside the UK)**	
0–2 years	4 (5.7%)
2–5 years	5 (7.2%)
5–10 years	10 (14.3%)
> 10 years	50 (71.4%)
Missing	1 (1.4%)
**Redeployed during COVID-19**	
Redeployed	22 (13.4%)
Change in work pattern	42 (26.8%)
Not redeployed	93 (56.7%)
Not applicable ^d^	7 (4.3%)

^a^Includes White British, Irish, Gypsy or Irish Traveller, Roma and White Other, as defined by the Office for National Statistics [33]. All participants’ ethnic groups were self-identified in our study

^b^Also includes dentists, pharmacists, healthcare scientists, ambulance workers and those in optical roles

^c^Includes those in administrative, or other non-clinical roles (e.g., housekeeping/ security/ maintenance, etc.)

^d^Includes HCWs who were either working in a private practice, self-employed, or shielding

**Fig. 1:**
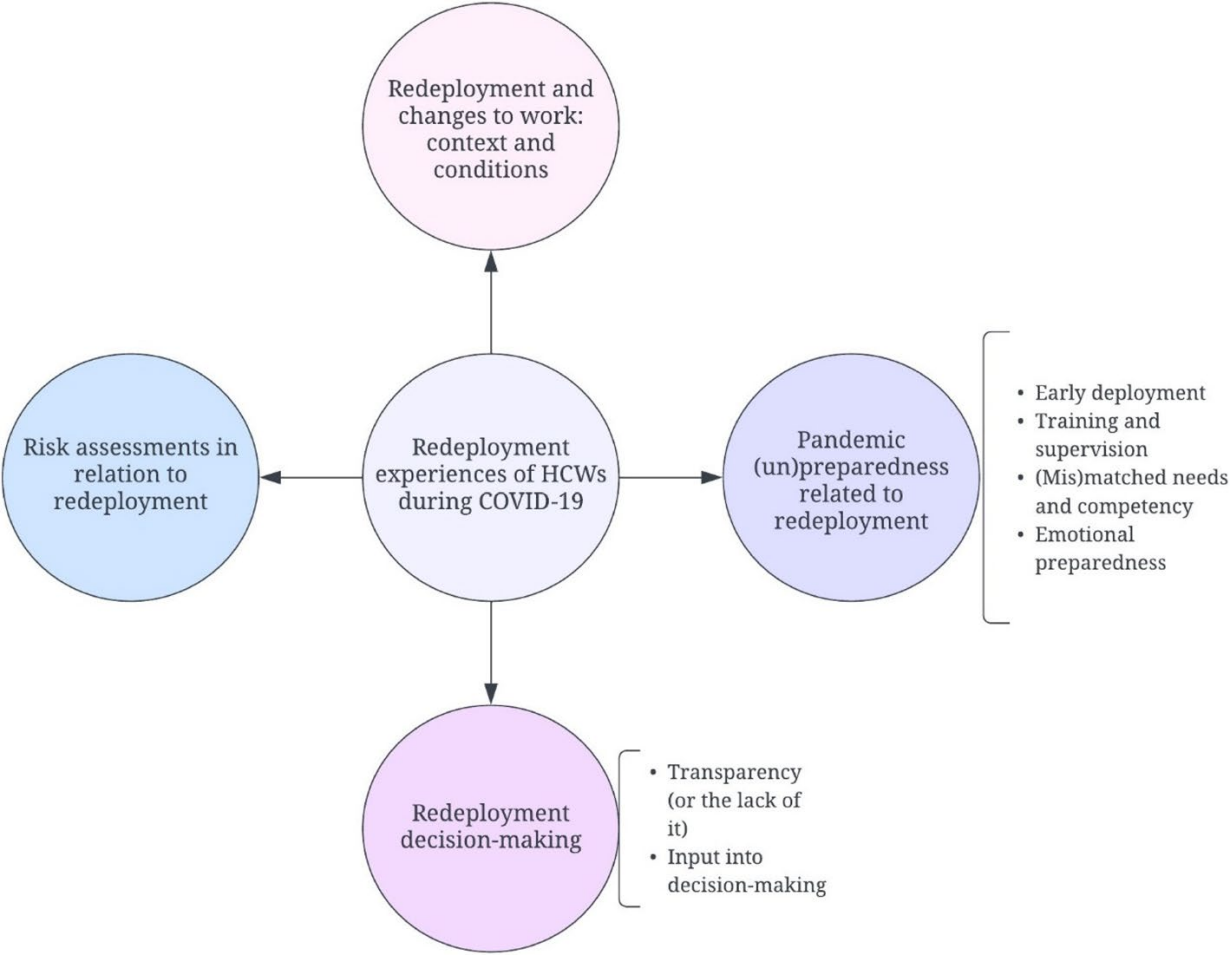
Redeployment experiences of HCWs during COVID-19—themes

There was variation in redeployment experiences among HCWs in our sample as decisions were made by employers on an ad-hoc basis in different locations and trusts, and HCWs attitudes, sentiments and perspectives about redeployment were largely dependent on what kind of experiences they had at their respective workplaces and with their working arrangements. Four major themes were developed which highlight (a) redeployment and the changing nature of work; (b) pandemic (un)preparedness related to redeployment; (c) redeployment decision-making and; (d) risk assessments in the context of redeployment. Each of these themes (and related sub-themes) are presented in detail below. A diagrammatic presentation of the themes and sub-themes is shown in Fig. 1.

### Redeployment and changes to work: context and conditions

Participants reported how within their workplaces, the surge in hospital admissions during the pandemic necessitated the deployment of HCWs into new roles and/or new settings to meet growing demands. Moreover, roles also had to be redefined on many occasions to account for staff absences due to COVID-19 related sickness and ‘shielding’ of vulnerable staff. For example, some HCWs pointed out that although they had not been redeployed in the strict sense of the term, the nature of their work changed significantly, requiring them to engage in tasks which were not part of their routine duty. As a medical registrar working in an acute hospital explained:

*“So I’ve not been contacted about redeployment but the role has changed in terms of the medical registrar at the [hospital]...So there’s usually only three medical wards there but in terms of this second wave [of the pandemic] now they’ve had to open up another additional medical ward. So just my work role in terms of what wards I’m covering and ward rounds...and what I do during the shifts has changed.” [Participant 1, Indian, Doctor]*

For most of the clinical staff, redeployment was mainly into COVID facing roles in Intensive Therapy Units (ITUs) and wards. A junior doctor who was redeployed and was presumably not able to receive her surgery training as a result mentioned:

*“...end of March, beginning of April time, I was due to move on to a different clinical rotation, because we rotate every quarter, I was meant to go to surgery but I never moved* *because of Covid so I was redeployed to Covid wards, that’s where I worked for four months, so yeah, it was like lots of random different medical wards that had been filled with obviously just Covid patients.” [Participant 2, Arab, Doctor]*

However, at times staff were also redeployed out of COVID roles/areas if they were deemed to be at risk. Speaking about colleagues who were redeployed due to their health risks, a HCW said:

*“We’ve got one chap who has underlying health conditions who has been moved into, he does quite a lot of security work for an agency, so he’s less patient facing now...Another chap that I’m aware of who has underlying health conditions...he’s just down in the equipment library delivering pumps, air mattresses and things like that. So he’s completely non-patient facing” [Participant 3, White, AHW]*

Redeployment also happened as certain specialities/services, that were regarded as non-urgent, closed or suspended, and staff had to be moved to other urgent service areas. A nurse, who was redeployed during the first wave of the pandemic shared:

*“The role changed immediately because we had to suspend all clinical access for patients immediately. We have an [Service], so the whole service was really, stopped very abruptly at the same time really for everybody, more or less on the same day, and then it was just a case really of us being redeployed into other areas. So most of the [speciality] staff were redeployed to other places. I was part of a small team that was redeployed to [name of place], into [speciality].” [Participant 4, White, Nurse]*

### Pandemic (un)preparedness related to redeployment

Results indicate that redeployment was one of the areas that lacked proper planning and preparedness. Many HCWs alluded that redeployment, particularly in the initial days of the pandemic, was done in an ad hoc manner which resulted in mismanagement, communication gaps, and confusion which made HCWs feel anxious and unsure of what they were expected to do. However, there were also those who felt that they were adequately prepared for their redeployed roles which led them to have a positive experience. In the following sub-themes, we detail this preparedness (or lack of it) in several aspects of redeployment such as early deployment, training and supervision, matching needs and competency and emotional preparedness of redeployed staff.

#### Early deployment

Some of our participants shared that they were given very short notice about their redeployment, sometimes just a day. In their own words:

*“So going in and being told that I was going to sort of, we knew it was coming our way. It was just really, really frightening and really scary. I wished that I had had a bit more notice to prepare going, but it was just going into work and our manager just said, “I’ve nominated you to go first” and that was sort of quite – it was unnerving you know. [Participant 5, British Asian, Staff Nurse]*

*“I was redeployed because they didn’t have anyone to do that job. They needed someone at short notice with these skills and I was identified as that person, so Monday I’m told and Tuesday I start.” [Participant 6, White, AHW]*

#### Training and supervision

Participants in our study mentioned varied experiences of training and supervision in their new roles. For some, the training they received was not sufficient to prepare them for the ground reality while some others reported that they were satisfied with the training and guidance they received. One redeployed nurse, who felt that the training fell short of preparing her for her new role in ITU shared:

*“So we had one day which we had to attend [a training], maybe half a day, if that, of sessions, preparations for coming to intensive care...but actually nothing prepares you for when you walk in that area...So I had this training on the Tuesday and then on the Wednesday I was there already but, to be fair, all that training just went off, you know, and what I felt helped me was my experience as a nurse through the years...but I felt had I been given the chance to actually visit the area, to actually put that PPE on me before I had to actually look after the patient, just put the PPE, just go and see the area on the next day probably I would have felt much better.” [Participant 7, Indian, Nurse]*

Speaking of the importance of training for redeployed staff and how the lack of proper training affected staff morale and anxiety during the pandemic, another HCW remarked:

*“So training is another one because I think the first wave...the staff were redeployed in areas that they don’t have any skill sets. So I think even though you don’t work in a ward, I think probably you need to train the staff...I think that the reason why they’re more upset is because they’re there but they don’t know what to do sometimes if they haven’t been in that kind of environment. So they feel that they’re not really ready to face the battle or on the battlefield.” [Participant 8, Other (Filipino), AHW]*

However, as previously mentioned, not everyone had the same experience and some redeployed HCWs appreciated the training they received and felt it was adequate to carry out COVID-19 specific duties.

*“I think it was very, the retraining and redeployment was adequate for our skills. It was not too much, asking too much of us so for example because we are [speciality], we don’t practise medicine normally so we were not asked to prescribe or examine medically ill patients. Our role was to help patients being, to turn patients on ITU who were on a ventilator, proning as it is called, and things like that”. [Participant 9, Arab, Doctor]*

Similar experiences were also shared by redeployed staff when it came to receiving supervision or support in their new roles. Speaking of the lack of supervision in their new role, one HCW remarked, “…*all through the time I was in that role, not once did I have any support from the line manager*” *[Participant 18, Indian, AHW].* The lack of orientation and support in her redeployed role was also mentioned by another HCW:

*I was sent with no job description, no structure, no governance, no rules, no nothing, just do something, but we didn’t know what, we didn’t know how, every day we had new meetings…I wasn’t given any support to succeed in that, wasn’t given any pay rise, any compensation, I was just told, you don’t know when you’re going to have a next day off. [Participant 6, White, AHW]*

#### (Mis)matched needs and competency

Our data show that most HCWs felt redeployment was not handled systematically, leading them to believe that valuable resources were wasted, or tensions were created between staff members. For instance, one participant described the frictions between redeployed nurses and existing ITU staff arising out of poor planning, stating:

*“It’s not the failing of the redeployed nurse, and not the failing of ITU, it’s the failing of planning. If they planned it properly then they could have, you know, do a little bit more of training just before they come in, and also designation – inform the ITU nurse if this is a nurse in the ward and had no ITU experience, just use them as a runner.” [Participant 10, Asian (Filipino), Nurse]*

Another HCW commented on how some HCWs were becoming redundant in their redeployed roles:

*“So we tried to redeploy a few people at the beginning because we were asked to and because it seemed like the right thing to do, but actually they were standing around doing nothing so we took them back again because actually we could use them better.” [Participant 11, White (Danish), Nurse]*

Speaking of the mismatch in redeployed HCWs’ competencies and their newly assigned roles, one HCW said:

*“So we actually did redeploy but we didn’t redeploy them to do the work that they were qualified to do, so there might be a senior registrar level almost at the end of the training so they were not redeployed to work at registrar level...They were all redeployed to work at very junior levels to support with some of the more regular clerking, trying to get the documents up to date, making sure that their temperature was taken, those kind of activities*.” *[Participant 12, Asian (Indian), AHW]*

While the lack of coordination and planning was pointed out by most HCWs, several of them also said that things improved as the pandemic progressed.

*“It’s the redeployment where staff go to the ward and help with the ward which they don’t usually do so it’s things like there’s overflowing of staff who is not doing anything, they’re just standing, wearing PPE unnecessarily, do you know what I mean? But then again that was the first wave but the second wave...it’s better handled.” [Participant 13, Asian (Filipino), Nurse]*

#### Emotional preparedness

Another aspect of preparedness relating to redeployment was the emotional burden that came along with working in new roles, new teams and for some HCWs in environments that they were not familiar with. For example, at a time when hospitals were witnessing mass deaths, a HCW who worked in administrative roles before the pandemic was redeployed to work in a mortuary. Speaking of this experience, the HCW said:

*“You were put into whatever you were capable of doing. So I came from a position of never being clinical, never seeing somebody that had passed away, because I literally, I actually had a fear of dead bodies before I went and worked in the mortuary...And within an hour of going down and helping, that had gone because you just couldn’t think like that anymore and it was – and is – overwhelming.” [Participant 14, White, Nurse]*

Another HCW in a redeployed role who was liaising with COVID-19 positive staff over the phone, spoke of the emotional demands of her new role:

*And then a lot of bad news as well, like there was a lady…she told me oh it was her husband that had symptoms, it wasn’t her and then I just went ‘oh OK so how is he now’ and then she went ‘oh he died yesterday’. And then I was like ‘what, he what’, then I just started crying on the phone like ‘I’m so sorry, I’m so sorry’…And so that’s kind of, like, this is something I was never exposed to before…changed my responsibilities a lot, because then I had to start doing things that I wasn’t trained or prepared to do but I had to do it anyway.” [Participant 15, Black, AHW]*

Additionally, HCWs in the study also said that because staff were being redeployed in haste, and due to the frequent turnovers, the sense of team was missing, which had an impact on workplace dynamics.

*“…when people were calling me and talking about their experiences when they were redeployed to other hospitals that’s not theirs kind of, like, treating them like second class citizens and that’s got nothing to do with anybody’s background, it’s just because I felt and the staff felt because they are not employed by that hospital they don’t see them as important as their staff.”[Participant 16, Asian (Indian), AHP]*

### Redeployment decision-making

#### Transparency (or the lack of it)

Most participants felt that there was a lack of transparency and clarity around the decision-making process for redeployment. This lack of transparency meant that HCWs were unsure how people were chosen for redeployment and this in turn created suspicions that certain groups were deliberately chosen for high-risk roles. In particular, some participants from ethnic minority groups themselves felt that HCWs from their own backgrounds were redeployed more compared to their White counterparts despite being at higher risks. Reporting on the discrepancies in identifying staff for redeployment at their workplaces, some HCWs shared:

*“when I see some of these staff that are working on the Covid ward and I think surely your risk assessment must be high, you should be on a non-Covid ward, but I just don’t know how they’ve arranged redeployment and things...” [Participant 17, Mixed, Doctor]*

*“I’ve seen staff that have been very tired, I don’t know how people have been selected for redeployment…” [Participant 18, Indian, AHW]*

*“So I was working in intensive care because I was in intensive care before I moved to recovery. So it’s always the BAME staff really that have been moved because we are from the [place 4] Hospital and we are sent to the Covid – like some of my colleagues are sent to the Covid wards or sent to intensive care in other hospitals. So I just noticed the pattern really.” [Participant 19, Asian (Filipino), Nurse]*

Additionally, some participants felt that the fear of repercussion also made it difficult for HCWs in certain situations, e.g. those on visas, working in lower paid or junior roles or those newly recruited, to question their redeployment decisions.

*“And I think that that is a structural – they were management decisions and I think people’s own unconscious bias and prejudice made them absolutely one hundred percent divert people of colour to those high-risk areas, because they’re not going to speak up, especially if you’re an immigrant and you want your job, you’re just not going to do it, it’s just not going to happen.”[Participant 20, British Asian, Nurse]*

Some participants also reported that because the processes were arbitrary, there were loopholes that allowed some HCWs to avoid being redeployment. For example, one participant remarked how in her institution, those with a good rapport with their supervisors or even those who falsified information, were able to avoid redeployment.

*“In terms of the rest of us, there was a bit of discrepancy, some people that very much were very friendly with their coordinators and things like that, sometimes got their way and made it clear that they didn’t want to be redeployed. And sometimes...there are others who have gone and told a story...who have never been probably fact checked, I guess because of the nature of like high alert of a pandemic, that have kind of just been sold ‘OK, fine, you don’t have to be redeployed’ kind of thing, but most of us were”[Participant 2, Arab, Doctor]*

#### Input into decision-making

Related to the lack of transparency is how some HCWs felt about being able to make their own decisions about redeployment. Several HCWs in our study mentioned that they had volunteered for redeployment, mainly out of a sense of duty. Although this may not have materialised in actual redeployment for everyone but having that sense of agency or control mattered about how they felt about the process. As one HCW mentioned:

*“…the good thing was with redeployed teams – they had volunteered and put themselves forward to come and do something while their services were stood down. That was at the beginning and that was absolutely the right way because the last thing we wanted was any nervous nellies. Later on when they were saying we need more staff to be doing the face fit testing, the staff were being volunteered to do it by their managers – now that was just – so I’d speak to the person themselves and, you know, they weren't suitable.”[Participant 21, White British, AHP]*

Sharing similar views, another HCW, who was redeployed by her manager said:

*“So I came across people that they were happy to be there, they volunteered to be there…I think when opportunities come then people should be given a choice, because this is like the 21st century, isn’t it, it’s not ‘I’m telling you, you’re doing it’, it shouldn’t be that kind of attitude.” [Participant 7, Indian, Nurse]*

It was not simply about decisions to be redeployed, some redeployed participants also felt that important decisions about their redeployment such as where and how long they would be redeployed for were also taken in a top-down manner without their involvement. One HCW shared,

*“There was one particular day that the hospital that I was redeployed to, the [place 1] Hospital from where I live is over an hour’s drive, and I went to work one day and I was told I wasn’t needed, that they had enough staff, and I had to take annual leave.” [Participant 5, British Asian, Nurse]*

Some participants also implied that perceived unfair redeployment could have had an adverse impact on the workforce, with dissatisfied staff leaving the workforce or reducing work hours:

*“…we lost a lot of staff that time, especially when the redeployment started, like, people who went into the community and then they were redeployed into the hospital, they’re like ‘oh I left the hospital for a reason, you can’t make me go back’, so they leave.” [Participant 17, Black, AHW]*

*“And it is because of this experience because I feel like I have – I’ve done two rounds of redeployment now…there is the third round of redeployment and I don’t want to be there. Our shift patterns have changed…without consultation and I just don’t want to be part of this team anymore, so I’ve opted to do bank work and agency, then I’ve got a bit more control over my time.” [Participant 5, British Asian, Nurse]*

On the contrary, some participants shared positive experiences of feeling empowered during redeployment. Those in managerial positions had input into redeployment decisions and were able to support high-risk staff in their roles.

*“I feel like I have been more empowered now based on my experience, so when I ask the HR, when I go to HR and to occ health and say ‘we need to support staff redeployment to go on lesser risk areas. If we want to keep this staff at work, if we want to support this person’s attendance at work, we need to support redeployment’. So I felt like definitely HR and occ health have empowered me to do that and they have listened with me.” [Participant 22, Asian (Filipino), AHW]*

### Risk assessments in relation to redeployment

Several participants reported that prior to their redeployment, there was inconsistency in conducting risk assessments. These assessments, essential for identifying personal risk factors and making necessary adjustments to work environments, were critical for minimising risks to both healthcare workers and patients. Those in managerial positions, responsible for conducting these assessments, admitted that they had to reassure employees that the assessments would occur, but failed to follow through.

*“as a manager, I was constantly picking up phone calls from individuals who were stressed and who felt that this is not what they had signed up for. But it was a matter of trying to maintain your cool I think and trying to say ‘sorry, I wish we could do something different but we will flag this up, we will make a note of this and we’ll record it and then get risk assessments in place’, but I must confess that risk assessments did not happen.”[Participant 12, Asian (Indian), AHW]*

Some participants also remarked that their risks, based on factors such as age and ethnicity, were disregarded when redeployed into COVID roles. Instead, they were simply advised to protect themselves with personal protective equipment (PPE). This lack of acknowledgment of risks, created disappointments among staff and they felt this exposed the underlying racism and discrimination within the system.

***“****I have worked hard for the Health Service and my life was not respected. And there is so much that I can do, I just can’t be with the Covid patients because I’m more at risk. And my age, my ethnicity, that alone are two factors that deem me higher risk, and it was just like “Well just wear your mask and you’ll be fine” you know. It was just a really, really, really horrific time, and I don’t mind participating in anything to put forward how horrific that is. I mean the situation itself was awful but the way it was dealt with was awful. No risk assessment, no acknowledgement that you’re at higher risk. And for me it just highlighted how racist the Health Service…” [Participant 20, British Asian, Nurse]*

The racism and lack of support became even more apparent for some HCWs when their managers neglected to take responsibility for seeking out guidelines regarding risk assessment in relation to ethnicity, before redeploying them.

*“[…] but they didn’t refer me to occupational health. And then in January we were redeployed again, and I then said to my manager that risk assessment that I had before in June was not BAME specific. You see, by January there was a lot more information about the effect on my [unclear] a lot more evidence. And she said “Oh well I’m not sure how it affects ‘ethnicals’. [Participant 20, British Asian, Nurse]*

For some of our participants, though, reasonable adjustments were made to their roles after their risk assessments found them to be at high risk. For example, a nursing assistant who was redeployed into another role after being unwell with COVID, mentioned,

*So after starting in the red ward and after having Covid, the stress was so high, I started getting migraines and having epileptic seizures…They did my risk assessment so they gave me an option to move to another wardso I said ‘OK, I’ll...’ [Participant 23, Indian, Nurse]*

## Discussion

This is one of the earliest studies conducted during the pandemic with the largest cohort of HCWs from diverse ethnic backgrounds and both clinical and non-clinical roles, offering valuable insights into their experiences. The redeployment analysis adds substantially to the limited evidence base on the topic, both by presenting new findings and substantiating existing ones. Firstly, our study has helpfully described what redeployment looked like on the ground, explicating the different context and conditions mentioned by our participants. Secondly, we were also able to capture the richness of experience of those who were not officially redeployed but their roles underwent significant changes. It is also important to note that our findings supplement the existing literature regarding inequities in redeployment practices, by showing how such practices can proliferate and disadvantage certain groups such as ethnic minorities, those on visas and/or, working in lower paid or junior roles, as these groups feel less empowered to question decisions made by higher authorities [10, 34]. In our knowledge, this is also the first analysis to show a link between redeployment, changes to working practices and staff attrition and hence can provide valuable insights to staff retention policies.

In terms of implementation of the relevant policy guidance [21] our findings suggest there were discrepancies between policy guidance and actual practice, especially in terms of early deployment, and fulfilling training and supervision needs. The lack of preparedness, both operational and emotional for HCWs had a detrimental impact on the HCWs involved in sudden and directed redeployment. The timescales for redeployment of some HCWs were as short as 24 h, which was in contravention of the relevant guidance and similar practices were reported in previous studies as well [35]. Timely redeployment has been included in the guidance because it has an impact on preparedness of HCWs for their new roles [21]. But when decisions are communicated at short notice, the opportunity to prepare and train staff is limited which might add to their anxieties. Additionally, as mentioned in other studies, HCWs in our study also expressed that not having clear direction, training and supervision in their redeployed roles increased their stress and anxiety [8, 36].

Existing evidence shows that redeployment, if organised strategically with clear communication and consideration for HCWs training and personal needs can be successful and have positive impacts on the workforce [35, 37, 38]. Or putting it otherwise, unplanned or poorly planned redeployment can have detrimental effects on staff morale and trust as reported by our participants. Chaotic, haphazard, and unplanned redeployment made many of them sceptical of the usefulness of it and thus it failed to capitalise on the high moral sense of duty that HCWs were feeling during the pandemic.

Our data also sheds new light on redeployment decision-making by reporting the role that transparency and agency played in the process. In our study, some HCWs felt they were part of the decision-making process around redeployment, with some even volunteering for redeployment or others opting out of it due to their perceived risks. This ability to take part in the process, demonstrate agency and engage with a transparent system for redeployment was much valued by those who could exercise this flexibility or benefitted from it. The autonomy and control that HCWs felt or desired about making redeployment choices was an important consideration reported in other studies as well [35, 39]. However, this opportunity to exercise autonomy was not available to all and non-transparent processes led to some HCWs feeling disempowered, and being suspicious of how these decisions were being made and to the detriment of certain groups.

This disparity could be attributed to systemic racism within the NHS, as existing evidence highlights how systemic inequities at institutional and individual levels disproportionately affect minoritised HCWs [40]. Migrant HCWs, for instance, may fear challenging decisions due to concerns about visa status or job security, exacerbating their vulnerability during redeployment [41].

Our data also supports the importance of team dynamics in redeployment and whether HCWs felt integrated or isolated in their redeployed roles [42]. Furthermore, not having proper risk assessments or not acknowledging people’s risks also contributed to some HCWs feeling that their safety and well-being were not valued [43]. This is concerning as we know there are existing issues around lack of trust for some minoritised communities and the systems they live and work in [7]. Our analysis shows that these experiences and perceptions can have consequences for the workforce, with some HCWs considering reducing their working hours or even leaving the profession altogether. This could have serious implications for the healthcare service which is already struggling to maintain adequate levels of staffing [18, 44].

### Relevance in today’s context

In the post-pandemic era, the NHS faces a substantial backlog of care worsened by severe staffing shortages, creating a demoralising cycle [18, 19]. Pre-existing challenges, such as staffing shortages, burnout, job dissatisfaction, excessive workloads, and racial inequalities, were worsened by the pandemic, particularly among nurses [45]. These compounded pressures, in addition to frequent redeployment of staff to different wards, have left HCWs feeling undervalued and disempowered [20]. In the current climate, where HCWs are overworked and experiencing pay erosion, this situation worsens staff retention, leading to a vicious cycle that hinders the delivery of quality care and diminishes job satisfaction [18–20], making it an important issue for leaders to address.

### Strengths and limitations

The UK-REACH qualitative sub-study was conducted to understand the experiences of a diverse cohort of HCWs’ during the pandemic and explored a range of topics including working conditions, risk perception, PPE, fears and concerns, and support and coping. Hence, the focus of the study was not specifically on redeployment experiences in its original design and therefore did not just include redeployed staff. This meant that the research question being analysed in this paper was not applicable to the full cohort. However, because the study had a relatively large sample, we have been able to draw out findings from the significant number of transcripts that discussed redeployment and meaningfully analyse these using the ‘breadth-and-depth’ approach.

Stemming from the previous limitation, we also realised that, because the focus of the study was not on redeployment, the management perspective from stakeholders such as HR managers, executive members, and team leaders, on how the process occurred and how decisions were made, were not uniformly collected. Despite this, we believe that understanding how HCWs experienced redeployment and their perceptions of what was or was not done correctly by decision-makers is important to inform future practice and policy on workforce planning and management.

Lastly, we acknowledge that there might have been a negativity bias [41] among our participants which might have predisposed them to share more negative experiences and perceptions than positive. We engaged in active reflexivity throughout the research process to minimise the potential for negativity bias. This included reflecting on our positionality and experiences to ensure we were not applying a negative lens influenced by our own social contexts. Additionally, we have tried to balance the findings by including, where appropriate, positive experiences of redeployment that some HCWs shared, aiming to provide a comprehensive and nuanced understanding of their experiences.

## Conclusions and recommendations

This qualitative analysis of redeployment experiences of HCWs in the UK presents findings from 164 HCWs from diverse ethnicities and working in both clinical and non-clinical roles. Our study adds significantly to the existing but scant literature on redeployment that happened during the COVID-19 pandemic. In accordance with previous studies, our study reiterates the importance of training and supervision for redeployed staff, particularly those redeployed to clinical roles. We, however, have added further evidence to show how transparency in decision-making, planning and preparedness could go a long way in creating trust, making staff feel valued and allow them to have fulfilling redeployment experiences. Importantly, our data also reveals important insights into how redeployment decisions could have influenced HCWs’ intentions to leave the workforce. These insights, although anchored in the pandemic and in redeployment, can make important contributions in workforce policy development, especially at a time when staffing levels have reached critical thresholds.

### Recommendations

In light of the evidence provided in this study and drawing from the literature, redeployment is an essential and important strategy for workforce management during crises, but to be effective, it has to: (a) take care of HCWs training and supervision needs; (b) be flexible to accommodate redeployed workers’ personal risks and situations; (c) be strategically planned and accompanied with adequate orientation and debriefing, not just for the redeployed staff but also for teams who are receiving redeployed workers; (d) have clear communication and support available for staff; (e) integrate an intersectional equity lens in workforce planning which acknowledges that systemic bias can result in disproportionate redeployment for certain HCWs [46, 47]; and (f) foster a sense of value and appreciation among HCWs. To improve the sense of value among staff, we recommend the following strategies, drawn from HCW feedback in one of our other studies:

1. Inclusion of minoritised staff groups in regular dialogue within senior management forums.
2. Flexible working arrangements to support and improve staff mental well-being.
3. Implementation of sustainable staff wellbeing initiatives.
4. Adopting more nuanced approaches to assess staff needs [44].

## Data Availability

The data for this study consists of interview transcripts of participants that contain potentially identifying and sensitive information. The data cannot be shared publicly due to concerns of participant confidentiality and ethics requirements. Participants consented to the study with the understanding that only de-identified quotations would be made public, not the entirety of the transcripts. Therefore, only illustrative quotes from the transcripts have been included in this paper. Data for this study could be made available upon reasonable request to the UK-REACH Data Access Committee (uk-reach@le.ac.uk), which is the institutional email of the UK-REACH project. For the purpose of open access, the author has applied a Creative Commons Attribution license (CC BY) to any Author Accepted Manuscript version arising from this submission, which permits others to copy, redistribute, remix, transform and build upon this work for any purpose, provided the original work is properly cited.

## Appendix 1

**Table 2:** List of keywords used while scanning data during breadth analysis

1. Redeployment
2. Risk assessment
3. Change in work patterns
4. Training
5. Supervision
6. Flexibility
7. Competency
8. Self-assessment
9. Induction
10. E-learning
11. Support

## Abbreviations

HCW: Healthcare Workers
NHS: National Health Service
UK-REACH: United Kingdom Research study into Ethnicity And COVID-19 outcomes among Healthcare workers
GMC: General Medical Council
NMC: Nursing and Midwifery Council
PPE: Personal Protective Equipment
HCPC: Health and Care Professions Council
GOC: General Optical Council
BAPIO: British Association of Physicians of Indian Origin
FNAUK: Filipino Nurses Association UK
PEP: Professional Expert Panel
STAG: Stakeholder Advisory Group
ITU: Intensive Therapy Unit
